# Impact of Status-Based Rejection Sensitivity on Depression and Anxiety Symptoms in Gay Men

**DOI:** 10.3390/ijerph17051546

**Published:** 2020-02-28

**Authors:** Joseph Slimowicz, Jedidiah Siev, Paula M. Brochu

**Affiliations:** 1 Private Practice, San Diego, CA 92109, USA; 2 Department of Psychology, Swarthmore College, Swarthmore, PA 19081, USA; jsiev1@swarthmore.edu; 3 Nova Southeastern University, College of Psychology, Fort Lauderdale, FL 33314, USA; pbrochu@nova.edu

**Keywords:** stress, psychological, LGBT persons, gays, anxiety, depression

## Abstract

Status-based rejection sensitivity refers to the anxious expectation and tendency to perceive rejection in ambiguous social scenarios based on one’s minority identification. This study evaluates the implications of sensitivity to rejection based on sexual orientation identity on negative mental health outcomes. Current minority stress models include rejection sensitivity as a factor that may contribute to adverse negative psychosocial outcomes in LGBT persons. This study evaluates the role of rejection sensitivity alongside demographically relevant predictors such as age, race, education, and level of sexuality disclosure in predicting the presence of significant depression and anxiety scores among a sample of gay men. Results indicate that rejection sensitivity, sexuality openness, and anxiety were significant predictors of depression symptoms, whereas age and depression were significant predictors of anxiety symptoms. This study supports the role of rejection sensitivity as a contributor to negative mental health outcomes among gay men, particularly as it pertains to internalizing mental health disorders.

## 1. Introduction

Gay men often experience a vast amount of psychosocial difficulties and, as a group, are at a greater risk to experience deleterious mental health outcomes compared to heterosexual persons [1,2]. Gay men experience higher rates of internalizing mental health disorders, such as depression and anxiety, relative to heterosexual people and lesbian women [3,4,5]. Furthermore, LGBT people experience higher rates of externalizing disorders such as substance use dependence, suicidal ideation, and self-harm behaviors than heterosexual individuals [5]. While a larger body of work has examined the influence of parental acceptance and rejection on gay men’s mental health [6,7], only a limited, but developing, body of work has examined the direct impact of status-based rejection sensitivity on depression and anxiety symptoms. Building from this emerging research base, the purpose of the present study is to examine the direct association between rejection sensitivity and symptoms of depression and anxiety among a community sample of gay men.

In an effort to explain and account for these observed mental health differences among sexual and gender minorities, two major theories have emerged within the literature. The minority stress model [8,9] posits that negative reactions from others and society based on immutable characteristics or identification with a stigmatized group results in increased stress on an individual. Exposure to increased and chronic environmental stress may explain ways in which LGBT individuals are at increased risk for a myriad of negative mental health outcomes.

Furthering the minority stress model, Hatzenbuehler [10] provided a more integrated and inclusive model explaining the specific processes by which exposure to stigma-related events and stress leads to increased risk for psychopathology. Within the psychological mediation framework, Hatzenbuehler [10] focuses on both general psychological processes (inherent in all individuals independent of sexual orientation) and LGBT group-specific processes that account for the disparities demonstrated within this population. General psychological processes refer to the psychological vulnerabilities shared among people (including non-minorities) that may give rise to distress and difficulties with regulation of affect such as emotional regulation, social isolation, and varying levels of self-esteem and helplessness. Group specific processes refer to proximal stressors such as internalized homophobia, levels of rejection sensitivity, concealment, and distal stressors including experiences with discrimination, victimization, and violence toward an individual based on one’s sexual orientation. Both general psychological vulnerabilities and group-specific processes may interact conjointly and increase the likelihood of an individual experiencing pathological psychological processes that give rise to both internalizing disorders such as depression and anxiety and externalizing disorders such as substance use.

One common specific-group process contained within both Meyer’s [8,9] model and Hatzenbuehler’s [10] mediation framework is rejection sensitivity. Notably, recent work by Feinstein (2019) [11] discusses how inclusion of rejection sensitivity in these models may extend and complement existing minority stress models. Rejection sensitivity is defined as the anxious expectation of rejection coupled with a tendency to readily perceive and interpret rejection in the ambiguous interpersonal behavior of others [12]. Importantly, rejection sensitivity is one potential outcome of living in a society that stigmatizes minorities, increasing the likelihood of rejection experiences and stress based on structural inequality [9,13]. In a meta-analysis of 75 studies, a moderate association between rejection sensitivity, broadly defined, and mental health outcomes was found [14].

Among the previous studies that evaluated the role and impact of status-based rejection sensitivity within minority stress models to date, status-based rejection sensitivity has been shown to be a significant predictor of depressive symptoms and social anxiety among gay men. In a sample of 467 gay men and lesbian women recruited online, Feinstein, Goldfried, and Davila [15] demonstrated significant path analytic associations with a small effect size between rejection sensitivity and measures of depression and anxiety. Furthermore, status-based rejection sensitivity mediated the association between social anxiety symptoms and experiences of discrimination. Cohen, Feinstein, Rodriguez-Seijas, Taylor, and Newman [16] assessed the role of rejection sensitivity as a transdiagnostic risk factor for internalizing disorders in gay men. Rejection sensitivity was associated with generalized anxiety, social anxiety, and posttraumatic stress symptoms but not with depression in a sample of gay and bisexual male university students. More recently, Sattler and Christiansen [17] found that rejection sensitivity and victimization experiences predicted mental health problems including symptoms of somatization, obsessive-compulsive disorder, interpersonal sensitivity, depression, anxiety, hostility, phobic anxiety, paranoid ideation, and psychoticism among gay and bisexual men in Germany.

The association between rejection sensitivity and mental health outcomes holds regardless of participant age, although some studies with a broader age range among participants demonstrate a negative association between rejection sensitivity and age [18]. The association between rejection sensitivity and mental health outcomes also tends to hold regardless of participant race [16] although racial diversity among samples is limited. Because of the inclusion of rejection sensitivity within both of the two minority stress models, better understanding the association between status-based rejection sensitivity and internalizing disorders among a community sample of gay men is warranted and would add to the present body of literature, particularly by taking into consideration the role of demographic covariates.

While other components of the minority stress model such as internalized homophobia and experiences of discrimination have been well established within the literature as being linked to problematic mental health outcomes and sexual risk behaviors [19,20], only the small body of work previously mentioned has evaluated the direct effects of status-based rejection sensitivity on internalizing mental health disorders among gay men. Minority stress models indicate that exposure to chronic environmentally linked events such as prejudice and stigma may lead to increased chronic stressors. This in turn may lead to difficulties in drawing upon and marshalling the necessary psychological resources to engage in adequate coping [9]. Only one study to date [16] has evaluated the association between status-based rejection sensitivity, depression, and generalized anxiety. Unlike this study by Cohen and colleagues, the current study evaluates these symptoms within a community-based sample more representative in age range and also samples from a broad geographic area in the United States. Additional studies with more representative samples evaluating rejection sensitivity on a range of internalizing symptoms are called for given the disparate prevalence rates of these disorders in the gay community.

In the current literature, the effects of age on internalizing symptoms within gay men are mixed. In a large-scale meta-analysis, age was demonstrated to be a moderator of internalized homophobia and mental health problems such as depression and anxiety with younger age individuals at increased risk during the early periods of sexual identity development [19]. It has also been associated with varying levels of positive affect and overall depressive symptoms [21,22]. Contrary to the meta-analytic results, in sample of 388 LGBT persons, increased rates of mood disorders were indicated in older gay men [23]. Similar to age, degree of disclosure and “outness” have shown mixed outcomes on the well-being of LGBT individuals with greater wellbeing associated with disclosure [24] versus potential for deleterious outcomes depending on the context of disclosure [25]. Given that age, sexual orientation disclosure, and degree of “outness” have been associated with mental health outcomes, we sought to include these variables as covariates while investigating the association between status-based rejection sensitivity and depression and anxiety symptoms.

The dynamics surrounding mental health outcomes in LGBT persons are complex. The impact of discrimination among ethnic minorities, in conjunction with LGBT identity, contributes to overall distress [26,27]. We hypothesized that status-based rejection sensitivity would be a significant predictor of both depression and anxiety symptoms in a community sample of gay men even when controlling for these other predictors of age, education, race and ethnicity, and sexuality openness.

## 2. Methods

### 2.1. Participants

This study was reviewed by an institutional review board (University of North Carolina Wilmington, Protocol #H809-132) prior to recruitment efforts of human participants. Participants were recruited at night clubs frequented by members of the LGBT community and at large gay community events such as equality marches and Pride events in Washington, DC, USA, Baltimore, Raleigh, and Atlanta. During the recruitment phase, participants were approached by the first author, provided with a brief background of the study, and given a business card with contact information and a SurveyMonkey link if they expressed interest in participating. Participants then accessed the research protocol from any location with Internet access and completed an online consent and the battery of measures.

Approximately 1000 business cards were handed out over a six-month period. A total of 242 participants accessed the online survey and provided consent for participation. Of these 242 participants, 12 identified their sexuality as other than gay and were subsequently excluded from further analyses. Of the remaining 230 participants, 83 withdrew after providing consent but before completing any of the main outcome measures, which represents an attrition rate of 36%. The final sample consisted of 147 gay men who ranged in age from 18 to 59 years (M = 34.90, SD = 10.56). The majority (85%) of the sample identified as White. With respect to education, 48% of participants reported having obtained a Bachelor’s degree or higher. See Table 1 for a comprehensive description of participant characteristics.

### 2.2. Measures

Rejection sensitivity. The Gay Rejection Sensitivity Questionnaire (GRSQ) [28] is a 14-item measure that evaluates sexuality-based rejection sensitivity. The measure poses hypothetical situations relevant to various social encounters gay men may commonly experience (i.e., “You are in a locker room in a straight gym. One guy nearby moves to another area to change clothes”) and prompts participants to evaluate their expectations of rejection based on their sexual identity (1 = *very unlikely* to 6 = *very likely*) as well as level of concern arising from the encounter (1 = *very unconcerned* to 6 = *very concerned*). Rejection sensitivity scores are calculated as the mean of the products of the expectancy of rejection and corresponding concern scores for each hypothetical situation and then divide by 14 (total number of items on the measure). Scores range from 1 to 36, with higher scores representing greater rejection sensitivity. The scale demonstrated good internal reliability within the present sample (α = 0.85).

Sexuality openness. Sexuality openness was assessed using a single item in which participants were asked, “How open about your sexuality are you?” Responses ranged on a scale from 1 (*sexuality orientation completely hidden) to* 7 (*completely open with others about sexual orientation*). This measure was adopted from the original Pachankis GSRQ development article [28].

Depressive symptoms. The DSM-oriented depression subscale of the Achenbach Self Report form [29] was used to evaluate symptoms of depression. The DSM-Depression subscale is composed of 14 items rated by participants on a 3-point scale (0 = *not true*, 1 = *somewhat or sometimes true*, 2 = *very true or often true*). Scoring was conducted using the ASEBA Ages 18–59 computer module Assessment Data Manager to obtain t-scores as instructed by the authors.

Anxiety symptoms. The DSM-oriented anxiety subscale of the Achenbach Self Report Form [29] was used to evaluate symptoms of anxiety. The anxiety subscale is composed of seven items that measure both cognitive and physiological symptoms on a 3-point scale (0 = *not true*, 1 = *somewhat or sometimes true*, 2 = *very true or often true*). Symptoms represented on the scale include symptoms of general anxiety (e.g., worries about future, fearful, nervous) rather than specific anxiety concerns such as social anxiety disorder. Scoring was conducted using the ASEBA Ages 18–59 computer module Assessment Data Manager to obtain t-scores as instructed by the authors.

## 3. Results

### 3.1. Preliminary Analyses

The skewness statistics for the DSM depression (M = 57.15, SD = 8.01, range 50–82) and DSM anxiety (M = 56.04, SD = 6.65, range 50–75) subscales departed from normality (1.08 and 0.89, respectively; SE = 0.21) and the Shapiro–Wilk tests of normality were significant, W (140) = 0.84 and 0.83, respectively; *p* > 0.001. Transformations of the data to correct for deviations from normality were attempted but were unsuccessful. Therefore, following the recommendations of Streiner [30] the outcome variables of interest were dichotomized to correct for non-normality. For both the depression and anxiety scales, a t-score of 50 represents sub-threshold or “absent” symptoms, whereas t-scores of 51 or greater indicate the presence of psychological symptoms [29]. Thus, scores of 50 were coded as 0 and scores of 51 or greater were coded as 1. For depression, 100 participants were coded as 1 and for anxiety, 108 participants were coded as 1. Descriptive statistics and inter-correlations between the variables are presented in Table 2 and Table 3, respectively.

### 3.2. Logistic Regression

To determine the predictive power of status-based rejection sensitivity on the presence of depressive symptoms, a logistic regression analysis was conducted. Rejection sensitivity scores were entered as a predictor along with the covariates of age, race, education, self-reported sexuality openness, and anxiety symptoms. As demonstrated in Table 4, rejection sensitivity scores emerged as a significant predictor for the presence of depressive symptoms. Furthermore, sexuality openness and anxiety symptoms were significant predictors as well, such that decreased levels of openness about one’s sexuality and the presence of anxiety symptoms predicted the presence of depression symptoms. Supporting our hypothesis, participants who reported greater sexuality rejection sensitivity were at greater risk for the presence of depression symptoms.

**Anxiety.** To determine the predictive power of status-based rejection sensitivity on the presence of anxiety symptoms, another logistic regression analysis was conducted. Rejection sensitivity scores were entered as a predictor along with covariate variables pertaining to age, race, education, self-reported sexuality openness, and depression scores. Contrary to our hypothesis, rejection sensitivity did not predict anxiety scores. However, as shown in Table 5, the presence of depression symptoms and age emerged as significant predictors for the presence of anxiety symptoms. Specifically, individuals younger in age and with higher levels of depression were more likely to report anxiety symptoms.

## 4. Discussion

The current study examines status-based rejection sensitivity as a predictor of depression and anxiety in a community-based sample of gay men while controlling for germane demographic and individual difference variables. The findings build on an existing body of established work by documenting rejection sensitivity and levels of outness as a predictor of depression. The data also indicated age and depression symptoms as predictors of anxiety with no contribution of rejection sensitivity to the overall model. Decreased self-reported outness was associated with depressive symptoms. An inverse relationship was noted between age and anxiety scores, indicating that younger participants were more likely to report anxiety symptoms.

When evaluating the association between rejection sensitivity and depressive scores, rejection sensitivity scores, outness, and anxiety emerged as a significant predictor of DSM depressive scores. No other demographic variables were found to be significantly associated with depression. Thus, it appears that status-based rejection sensitivity demonstrates utility in accounting for the variance in depression scores pertaining to depression in gay men. This finding is consistent with prior research that examined the association between status-based rejection sensitivity and depression. Moreover, the findings generalize to a broad sample of gay men and are replicated while using alternative instrumentation to measure depression.

Gay men who experience higher levels of status-based rejection sensitivity may be at increased risk for a myriad of behaviors that may influence and contribute to the development of depressive symptoms. Previous research by Pachankis, Goldfried, and Ramrattan [28] demonstrated that behaviorally, status-based rejection sensitivity was associated with a decrease in assertive behavior. For example, rejection sensitivity among gay and bisexual men is a risk factor for condomless sex [31]. Furthermore, the existing literature has demonstrated that decreases in assertive behavior is associated with increased depressive symptoms [32,33]. Extending on past and current findings, one may theorize that individuals higher in status-based rejection sensitivity may be vulnerable to other behavioral and cognitive features that contribute to the development of depression, thus leading to an association between status-based rejection sensitivity.

Behaviorally, high rejection sensitive gay men’s decreased assertiveness may also decrease the likelihood of successfully navigating situations during which sexual minority individuals are exposed to stigma and prejudice [28]. This decrease in ability to navigate situations related to exposure to stigma and prejudice may also decrease beliefs in self-efficacy to navigate other challenging situations in one’s life. Furthermore, status-based rejection sensitivity and exposure to stigma and prejudice may also combine to create other behavioral manifestations such as an increase in social isolation, thus leading to decreases in available social support. This postulation is consistent with the finding that decreased levels of outness is associated with depressive symptoms. Thus, gay men who have not disclosed sexual identity status may have less available social support.

This decrease of social support, in turn, may lead to decreased abilities in managing other life stressors that give rise to depressive symptoms. Cognitively, individuals who possess higher levels of status-based rejection sensitivity, coupled with exposure to minority stress events, may experience and incorporate negative beliefs into schemas that manifest depressive cognitions [10,28]. The mechanisms associated with specific cognitions and behaviors that provide an explanation of this established relationship represents an area for additional research.

Based on prior research indicating that rejection sensitivity is associated with higher levels of social anxiety in gay men [16], it stands to reason that this association would generalize to a range of anxiety symptoms. We hypothesized this, considering that high rejection sensitive gay men may experience increased vigilance towards perceived social threat cues and rejection. In turn, this process might generalize and cause misinterpretation and overestimation of the potential for catastrophic and threatening events across life domains, thus increasing general symptoms of anxiety. The present findings and data, however, do not suggest an association between anxiety and rejection sensitivity, despite previously being supported by Cohen et al. [16]. One possible explanation for the differences found between this study and Cohen et al.’s [16] may be related to the differing compositions of the participants’ ages within the two samples. Cohen et al. [16] used a sample comprised solely of university students, while our sample used a community sample that provided a broader representation of age than in Cohen et al.’s [16] sample. If this association is dependent on age, there may have been reduced power to detect the association between rejection sensitivity and general symptoms of anxiety within our sample. This postulation is further supported by the additional finding within our study that anxiety symptoms are associated with younger age.

Furthermore, although age was hypothesized to be a demographically-relevant predictor, the specific direction of this relationship was not predicted. Because of inconsistent findings regarding the role of age on mental health outcomes, the association between age and anxiety found in this study requires further elucidation. Age may serve as a proxy variable for other relevant factors not measured in this study. For example, age may be related to strength of LGBT community support, as older individuals have had more time to synthesize their identity and create meaningful social connections which may buffer stressors associated with LGBT identity [21]. As such, young adulthood may be a developmentally vulnerable time period in which younger gay men are at increased risk for anxiety due to rejection sensitivity and other minority stressors. Furthermore, younger age may also be related to higher levels of anxiety symptoms in younger gay men due to the intersecting demands of early adulthood and building an integrated identity as gay men. Further understanding of cohort and intergenerational differences between gay men is needed. Alternatively, one might expect older age in to be associated with increased distress due to increased likelihood of exposure to more profound systemic discrimination; however, this postulation does not stand based on the findings within the current study. Future research may benefit from assessing how minority stressors change and manifest differently over time in the lives of gay men and the role of cohort effects on minority stress.

While the present study merely tested the direct associations between status-based rejection sensitivity, depression, and anxiety symptoms, the data provide further impetus for additional investigation of the specific processes by which these symptoms arise. Future research may benefit by investigating the role of other variables within the model such as group-specific and general psychological processes. For example, evaluating mediators and moderators of the outcomes between rejection sensitivity and depressive symptoms would allow for better understanding of factors to target within a therapeutic process.

### Limitations

While the present study provides additional data supporting the association between status-based rejection sensitivity and internalizing disorders, there are several limitations to the study. First, due to skewness of our main outcome variables which violated assumptions of normality, we dichotomized the variables measuring depression and anxiety. Unfortunately, this likely limited the range of variability of these measures, limiting our ability to detect significant associations with rejection sensitivity.

In reference to sampling methodology, the study drew its sample from a convenience-based sample and is subject to self-selection bias. Participants were recruited from gay bars and large gay community events, and so may score lower on rejection sensitivity and higher on sexuality openness than gay men who do not attend such community organizations or events. Gay men higher in rejection sensitivity may be more inclined to avoid the recruitment locales utilized in this study (e.g., gay bars). As a result, the current sample may be less generalizable to the gay population. The present sample may not be fully representative of a broad range of gay men, for example, those who live in more rural areas without access to local gay community life. Individuals who live in rural areas may experience greater levels of potential for discrimination and less social support from their environment. While this sample included a variety of geographic areas, this sample was most representative of gay men living in major metropolitan areas. Furthermore, the results are not generalizable to other sub-populations in the LGBT community such as lesbian woman, bisexual individuals, or transgender people as the sample was intentionally limited to gay men. It is possible that these associations may not be robustly applied to other populations as the concept of intersectionality has often demonstrated nuanced differences in the experiences of gay, lesbian, bisexual, and transgender communities [34,35].

Lastly, this study does not incorporate other relevant variables that may be associated with depression or anxiety in a sample of gay men (e.g., internalized homophobia, perceived discrimination) and instead evaluates the predictive utility of rejection sensitivity scores alongside relevant demographic variables. Meyer [13] argues that researchers must consider the social context from which rejection sensitivity originates, such as the experience of prejudice and discrimination. Furthermore, Meyer cautions against the misapplication of rejection sensitivity as describing people who are “overly sensitive” to stigma and discrimination, and pathologizing what is a normative response to structural inequality. Rejection sensitivity research would benefit from greater recognition that the stigmatizing social context in which people live is what causes minority stress and increases the risk for rejection sensitivity. This involves consideration of both individual and environmental factors that are related to minority stress, placing emphasis on reducing stigma and discrimination at the societal level [13]), as well as promoting coping with rejection sensitivity due to experiences of discrimination [15]. There is value in studying rejection sensitivity as a unique individual difference variable that may have utility in developing applied, empirically-based interventions to reduce the deleterious effects of exposure to structural stigma. However, it is important to also work to reduce the stress from stigma, discrimination, and actual experiences of rejection [13]. From a public health perspective, attention must be given to both environmental and individual factors to address the negative effects of stigma on marginalized communities at risk.

## 5. Conclusions

While several of the aforementioned limitations represent shortcomings, the present design allows for greater understanding of the association between status-based rejection sensitivity and outcomes important to the mental health concerns of gay men. Furthermore, it provides the impetus for further exploration of issues pertaining to the role of status-based rejection sensitivity within existing minority stress models. Current science in this area continues to grow and more refined models are produced to understand the etiology, causes, and implications for LGBT individuals’ exposure to minority stress. Future development and strengthening of these models have begun to lead to the development of empirically derived clinical interventions that may serve to buffer and decrease negative outcomes and distress associated with exposure to systematic stigma, oppression, and inequality faced by the LGBT community [36]. The current findings support future interventions that incorporate status-based rejection sensitivity as one factor that might be addressed in the therapeutic process to decrease depressive symptoms among gay men.

## Figures and Tables

**Table 1:** Study participants.

Variable	*n*	%
*Race*		
White/Caucasian	128	87
African American/Black	5	3.4
Hispanic/Latino	8	1.4
Asian/Pacific Islander	3	2.1
Other	3	2.0
*Education*		
No High School	2	1.4
HS Diploma	5	3.4
Some college	29	19.7
Associates Degree	5	3.4
Bachelor’s Degree	45	30.6
Some Graduate School	13	8.8
Master’s Degree	30	20.4
Doctoral Degree	14	9.5

**Table 2:** Statistics.

Variable	M	SD	Min	Max
Rejection Sensitivity	10.55	5.60	2.14	29.93
Depression	0.71	0.45	0	1
Anxiety	0.77	0.42	0	1
Education	0.75	0.44	0	1
Age	34.90	10.56	18	59
Race	0.13	0.34	0	1
Sexuality Openness	5.88	1.13	1	7

Note. Depression, anxiety, education, and race are dichotomized variables scored as follows: For depression, 0 = symptoms not present, 1 = symptoms present; for anxiety, 0 = symptoms not present, 1 = symptoms present; for education, 0 = no college degree, 1 = college degree; and for race, 0 = white, 1 = non-white.

**Table 3:** Correlations Between variables.

Variable	1	2	3	4	5	6	7
1. Rejection Sensitivity	1	0.20 *	0.13	0.08	0.02	−0.02	0.02
2. Depression	-	1	0.26 **	−0.02	−0.04	−0.04	−0.18 *
3. Anxiety	-	-	1	−0.24 **	−0.04	0.11	−0.08
4. Age	-	-	-	1	0.20 *	−0.01	0.22 **
5. Education	-	-	-	-	1	0.03	0.04
6. Race	-	-	-	-	-	1	−0.06
7. Sexuality Openness	-	-	-	-	-	-	-

Note. Depression, anxiety, education, and race are dichotomized variables scored as follows: For depression, 0 = symptoms not present, 1 = symptoms present; for anxiety, 0 = symptoms not present, 1 = symptoms present; for education, 0 = no college degree, 1 = college degree; and for race, 0 = white, 1 = non-white. * *p* < 0.05. ** *p* < 0.01.

**Table 4:** Logistic regression analysis on the presence of depression symptoms.

Variable	B	SE	Wald	*p*	OR
Anxiety	1.28	0.47	7.41	0.006	3.61
Age	0.02	0.02	0.48	0.488	1.02
Race	−0.29	0.60	0.23	0.629	0.75
Education	−0.31	0.50	0.37	0.541	0.74
Sexuality Openness	−0.45	0.22	4.18	0.041	0.64
Rejection Sensitivity	0.08	0.04	4.09	0.04	1.09

**Table 5:** Logistic regression analysis on the presence of anxiety symptoms.

Variable	B	SE	Wald	*p*	OR
Depression	1.28	0.48	7.28	0.007	3.61
Age	−0.05	0.02	6.19	0.013	0.95
Race	0.98	0.81	1.47	0.225	2.66
Education	0.20	0.53	0.15	0.699	1.23
Sexuality Openness	0.07	0.21	0.10	0.755	1.06
Rejection Sensitivity	0.06	0.04	1.66	0.196	1.06
